# A Pilot Study on the Use of Pumpkin Waste as Cattle Feed

**DOI:** 10.3390/metabo15080511

**Published:** 2025-07-31

**Authors:** Minori Nizuka, Hironobu Ishihara, Jun Nakahigashi, Daisaku Matsumoto, Eiji Kobayashi

**Affiliations:** 1 Wellness Development Center, Air Water Inc., 1-7, Tsukisamu Higashi 2-jo 16-chome, Toyohira-ku, Sapporo 062-0052, Hokkaido, Japan; 2 Air Water Logistics Co., Ltd., 1-6, Tsukisamu Higashi 2-jo 16-chome, Toyohira-ku, Sapporo 062-0052, Hokkaido, Japan; 3 Shepherd Central Livestock Clinic Co., Ltd., 2090-1 Akasegawa, Akune-shi 899-1611, Kagoshima, Japan; 4 Kobayashi Regenerative Research Institute, LLC, 1 Chayano-cho, Wakayama-shi 640-8263, Wakayama, Japan

**Keywords:** β-carotene, cattle feed, heptachlor, pumpkin seed

## Abstract

**Background/Objectives**: Pumpkin seed pulp from processing plants offers high nutritional value due to its rich β-carotene content, making it a potential functional feed ingredient. This study investigated the effects of pumpkin seed pulp, which has already been administered as livestock feed, on key physiological parameters in cattle, including the concentration of β-carotene in the blood measured during routine health monitoring. **Methods**: Here, pumpkin waste cultivated in various fields was processed into cattle feed (pumpkin seed pulp flakes, PSPFs) by grinding and drying, and residual pesticide (heptachlor) and β-carotene contents were measured. A pilot feeding trial was conducted with 13 cattle (7 in the treatment group and 6 in the control group) and blood component analysis was performed, and findings were contextualized with a literature review. **Results**: Heptachlor concentrations varied depending on the cultivation site of raw pumpkins. Among the six lots produced using raw materials sourced from fields not contracted by the Air Water Group—a collective of companies in which Air Water Inc. holds more than 51% ownership—three exceeded the regulatory limits for animal feed established in Japan. PSPFs contained high levels of β-carotene, as expected. Blood tests before and after the feeding trial indicated absorption of β-carotene in the cattle. Maintaining high plasma β-carotene concentrations in cattle has been associated with improved immune function and reproductive performance. **Conclusions**: Our study demonstrates that PSPFs are a promising, environmentally friendly, and natural β-carotene-rich feed ingredient. Tracing the cultivation fields of raw pumpkins can help ensure feed safety.

## 1. Introduction

β-carotene is a type of pigment known as a carotenoid, recognized for its antioxidant and mucosal protective properties [1,2]. It is also referred to as provitamin A because it is converted into vitamin A in the small intestine [3]. Cattle, among even-toed ungulates, are unique in their ability to absorb and accumulate β-carotene, and a correlation between blood β-carotene levels and reproductive performance has been reported [4,5,6,7,8,9,10,11,12]. Fresh pasture, a common feed for cattle, contains high levels of β-carotene, enabling cattle to supplement this nutrient through their diet. However, the increasing use of preserved feed, such as silage and hay, has led to concerns about insufficient dietary intake of β-carotene [13,14,15]. Consequently, health issues related to β-carotene or vitamin A deficiency in livestock have become a concern, prompting the addition of vitamin supplements to feed.

In Japan, the total production volume of pumpkins was approximately 182,900 tons in 2022, with Hokkaido accounting for approximately 94,000 tons [16]. Inedible parts, such as seeds and stringy pulp, are typically discarded and not effectively utilized. According to the Standard Tables of Food Composition in Japan, approximately 10% of *Cucurbita maxima*, a pumpkin variety commonly consumed in Japan, is discarded as inedible parts [17]. Pumpkin seeds are rich in functional components, such as fatty acids, proteins, and β-carotene, making them promising candidates for use in processed foods for humans and as livestock feed [18,19,20,21,22].

A recurring concern associated with pumpkins is the detection of heptachlor, a residual pesticide. Heptachlor is an organochlorine compound that was registered as a pesticide in Japan in 1957, but its registration was revoked in 1972 under the Agricultural Chemicals Control Act [23]. Globally, its production and use were banned under the Stockholm Convention on Persistent Organic Pollutants in 2001 [24]. Despite this, heptachlor is highly persistent in the environment and can remain in soil for extended periods. It is also selectively absorbed by cucurbitaceous plants, such as pumpkins [25,26,27]. As a result, even more than 50 years after its ban in Japan, heptachlor can still be concentrated in pumpkins grown in contaminated soil. There is concern that the drying process of pumpkin seeds and pulp, intended to enhance their utility as animal feed, may potentially lead to the concentrations of heptachlor residues absorbed by the pumpkin. This raises the possibility that the final product could exceed national regulatory limits for pesticide residues [28]. Heptachlor can accumulate in cattle fat tissue, and cases of heptachlor exceeding regulatory limits in beef have been reported, prompting the establishment of a maximum residual limit for feed in Japan (0.02 μg/g) [29,30]. The residue limit for heptachlor is set as the sum of the main metabolites of heptachlor, cis-heptachlor epoxide, and trans-heptachlor epoxide.

The present investigation aimed to demonstrate the potential of pumpkin seeds and fibrous pulp, which are discharged as by-products from pumpkin processing plants, as a source of β-carotene for livestock. To support the design of a future large-scale feeding trial, we analyzed the effects of pumpkin seed pulp, which is already used as a dietary supplement in cattle feed, on key physiological parameters in cattle.

## 2. Materials and Methods

### 2.1. Production of Pumpkin Seed Pulp Flakes (PSPFs)

Pumpkin residue (seeds and stringy pulp) discharged from the production line of a food processing facility was collected. The residue varied in size, ranging approximately from 20 to 100 mm. Its initial moisture content was 80.7% by weight. The collected residue was processed using a cutting device (URSCHEL Corp., Chesterton, IN, USA), operated at 3000 rpm for 60 min. This procedure reduced the residue, including the seeds, to fragments approximately 1–5 mm in size. The complete destruction of pumpkin seeds during processing serves as a precautionary measure to eliminate unintended pumpkin growth resulting from undigested seeds that could remain in cattle excreta. The resulting material was then dried using a vacuum dryer set to an internal temperature of 85 °C. The drying process was conducted for 16 h under conditions of 5 rpm agitation and an absolute pressure of 8.0–10.0 kPa. The treatment yielded a processed feed product derived from pumpkin residue, with a final moisture content of 10.8% by weight and particle size of approximately 1–5 mm. This processed feed has been patented in Japan (Patent No. 7506974) [31].

### 2.2. Analysis of Heptachlor Content

Heptachlor analysis was performed by an external company, Japan Food Research Laboratories (Tokyo, Japan), using an established protocol.

First, 10 g of the sample was combined with 30 mL of water and 100 mL of acetone, and subjected to shaking extraction for 60 min. The extract was filtered under vacuum, and the filtrate was adjusted to 200 mL with acetone. A 20 mL aliquot of this solution was concentrated and loaded onto a solid-phase extraction cartridge (InertSeo K-solute Plus, 5 mL; GL Sciences Inc., Tokyo, Japan), followed by elution with 40 mL of hexane. The eluate was concentrated to dryness and reconstituted to 10 mL with hexane. A 5 mL portion of this solution was then applied to a second solid-phase cartridge (Sep-Pak Plus Florisil, 910 mg; Waters Corporation, Milford, MA, USA), and eluted with 20 mL of a hexane–diethyl ether (24:1) mixture. The eluate was again concentrated to dryness and reconstituted in 1 mL of hexane. A 1 μL aliquot of this final solution was injected into the GC-MS.

GC-MS analysis was performed using a VF-5MS column (15 m in length, 0.25 mm inner diameter, and 0.25-μm thick film; Agilent Technologies, Santa Clara, CA, USA) in conjunction with an Agilent 7890B/7010B GC-MS system (Agilent Technologies, Santa Clara, CA, USA). The initial oven temperature was set at 70 °C and held for 1 min, then increased to 200 °C at a rate of 25 °C/min, followed by a ramp to 310 °C at 15 °C/min, and held for 5 min. The carrier gas (He) flow rate was maintained at 1.701 mL/min. The injector temperature was set to 250 °C and ion source temperature to 320 °C. Injection was performed in pulsed splitless mode (pulse pressure 25 psi, 1 min). Electron impact ionization was used to acquire mass spectra. Quantification was based on the peak areas of the mass fragments at m/z 272→237 (heptachlor), 355→265 (cis-heptachlor epoxide), and 217→182 (trans-heptachlor epoxide).

### 2.3. Nutritional Composition Analysis of PSPFs

Nutritional composition was analyzed by the Agricultural Chemistry Research Institute of the Tokachi Federation of Agricultural Cooperatives (Hokkaido, Japan) and was conducted in accordance with the Quality Evaluation Guidebook for Roughage, 3rd Edition, published by the Japan Grassland Agriculture and Forage Seed Association (Tokyo, Japan) [32]. Fatty acid analysis was outsourced to Japan Food Research Laboratories and conducted according to their established protocol.

For fatty acid analysis, 0.5 g of sample was mixed with 20 mg of heptadecanoic acid as an internal standard and 3 mL of a 1% sodium chloride solution containing 10% pyrogallol. The mixture was heated at 100 °C for 5 min. Subsequently, 1 mL of 66% potassium hydroxide solution and 10 mL of ethanol were added, and the mixture was heated at 70 °C for 30 min. Fatty acids were extracted twice with 15 mL of a mixture of ethyl acetate and hexane (1:9). Approximately 5 mL of the extract was collected, and the solvent was removed. Then, 1.5 mL of 0.5 mol/L sodium hydroxide in methanol was added, and the mixture was heated at 100 °C for 9 min. After adding 2 mL of boron trifluoride-methanol complex solution, the mixture was heated at 100 °C for 7 min. Finally, 3 mL of hexane and 5 mL of saturated saline were added, and the mixture was shaken. A 1-μL aliquot of the hexane layer was injected into the Gas Chromatography (GC). GC analysis was performed using an Agilent 7890B GC system (Agilent Technologies) equipped with a flame ionization detector and a DB-23 column (30 m length, 0.25 mm inner diameter, 0.25 μm film thickness; Agilent Technologies). The oven temperature was initially set at 50 °C and held for 1 min, then increased to 170 °C at a rate of 10 °C/min, followed by an increase to 210 °C at a rate of 1.2 °C/min. The carrier gas (helium) pressure was maintained at 70 kPa. The injector and detector temperatures were set at 250 °C, and the sample was injected with a split ratio of 1:20. The detector gas flow rates were set at 35 mL/min for hydrogen, 350 mL/min for air, and 20 mL/min for nitrogen as the makeup gas. Quantification was based on the peak areas obtained.

### 2.4. Analysis of β-Carotene

A β-carotene standard (purity ≥ 95.0%) was purchased from Kanto Chemical Co., Inc. (Tokyo, Japan). Pyrogallol (FUJIFILM Wako Pure Chemical Corporation, Osaka, Japan), hexane, acetone, ethanol, and toluene (Kanto Chemical Co., Inc.), potassium hydroxide (Junsei Chemical Co., Ltd., Tokyo, Japan), sodium chloride (Kanto Chemical Co., Inc.), ethyl acetate (Kanto Chemical Co., Inc.), and tetrahydrofuran (Junsei Chemical Co., Ltd.) were of Japanese Industrial Standards grade. The 2,6-Di-tert-butyl-p-cresol (Kanto Chemical Co., Inc.) was of Cica Special Grade, dl-α-tocopherol (FUJIFILM Wako Pure Chemical Corporation) was of Wako 1st Grade, and ethanol and cyclohexane (Junsei Chemical Co., Ltd.) were of HPLC grade. Acetonitrile (Kanto Chemical Co., Inc.) and acetic acid (FUJIFILM Wako Pure Chemical Corporation) were of LC-MS grade.

β-carotene was quantified using HPLC [33,34]. A 0.5 g powdered sample was accurately weighed into a brown volumetric flask, followed by the addition of 3 g of pyrogallol and 5 mL of water, and gently mixed. Then, 10 mL of HEAT solvent mixture (hexane/acetone/ethanol/toluene = 10/7/6/7 (*v*/*v*/*v*/*v*), containing 0.1% 2,6-Di-tert-butyl-p-cresol) was added and vigorously mixed. This step was repeated twice with 10 mL of the same HEAT mixture. Subsequently, 10 mL of ethanol containing 0.05 g/L α-tocopherol was added and vigorously mixed, and this step was repeated once. The solution was brought to volume with the same ethanol solution, sonicated for 10 min, and left to stand overnight.

After standing, 10 mL of the extract was transferred to a 50 mL tube and 2 g of potassium hydroxide was added. After vortexing, the mixture was saponified at 70 °C for 30 min, then rapidly cooled with water. A 20 mL portion of 1% (*w*/*v*) sodium chloride solution and 15 mL of a hexane/ethyl acetate mixture (9/1) were added, and the tube was shaken for 5 min under light shielding with aluminum foil. After centrifugation (2500 rpm, 2 min), the upper layer was transferred to a concentration vessel. The remaining lower layer was extracted twice more with 15 mL of the same solvent mixture. The combined upper layers were concentrated under reduced pressure at 40 °C, transferred to a 10 mL test tube while washing with the same solvent, and dried under nitrogen while warming at 40 °C. The residue was reconstituted to 5 mL volume in ethanol. A 10 μL aliquot of this solution was injected into an HPLC system (Agilent 1200 series, Agilent Technologies).

The analytical column used was Kinetex C18 (4.6 × 150 mm, 5 μm; Phenomenex, Torrance, CA, USA). The mobile phase consisted of acetonitrile/methanol/tetrahydrofuran/acetic acid = 55/40/5/0.1 (*v*/*v*/*v*/*v*) containing 0.05 g/L of dl-α-tocopherol and the flow rate was set at 0.8 mL/min. The column temperature was maintained at 40 °C and detection was performed at 455 nm. A blank solution prepared in the same manner without the sample was used as a control.

The β-carotene standard stock solution (100 μg/mL) was prepared in cyclohexane and diluted 50-fold to 2 μg/mL. The absorbance of this diluted solution was measured at 455 nm and the concentration was calculated using the following formula:

Concentration of standard solution (μg/mL) = absorbance at 455 nm × 10,000/2500(1)

Additionally, a 10-μg/mL standard solution was prepared by diluting the 100 μg/mL stock solution 10-fold with ethanol and further diluted to prepare a calibration curve in the range of 0.1–2 μg/mL.

### 2.5. Feeding Trial in Cattle (Pilot Study)

Seven adult Japanese Black donor cows were fed 500 g/day of pumpkin seed pulp flakes (PSPFs) in addition to their conventional silage-based diet for a period of 34 days. As a control group, six adult Japanese Black donor cows were maintained on the conventional diet alone. The amount of conventional feed provided to the control group was adjusted to match the dry matter intake of the treatment group. Blood samples were collected from both the treatment and control groups before and after the feeding period. Plasma was separated by centrifugation and subjected to biochemical analysis. The feeding trial was conducted at the donor cow facility of AG Embryo Support Co., Ltd. (Obihiro, Hokkaido, Japan). Blood biochemical analyses were outsourced to Obihiro Clinical Laboratory Inc. (Obihiro, Japan).

Blood biochemical parameters were evaluated, including glutamic oxaloacetic transaminase (GOT), gamma-glutamyl transferase (GGT), total cholesterol (T-CHO), total protein (TP), albumin (ALB), albumin/globulin ratio (A/G ratio), blood urea nitrogen (BUN), glucose (GLU), non-esterified fatty acids (NEFA), calcium (Ca), phosphorus (IP), magnesium (Mg), 3-hydroxybutyrate (3-HB), vitamin A (VA), and β-carotene (βC).

General biochemical blood tests were performed using the AU680 automated analyzer manufactured by Beckman Coulter, Inc. (Brea, CA, USA). The analysis of β-carotene and vitamin A concentrations in blood was conducted by HPLC with an established protocol [35,36].

### 2.6. Statistical Analysis

Statistical analyses were performed using Microsoft Excel (Microsoft Corporation, Redmond, WA, USA). For each blood biochemical parameter, the homogeneity of variances between pre- and post-administration values was assessed using the F-test at a significance level of 0.05. Student’s *t*-test was applied for parameters with equal variances, while Welch’s *t*-test was used for those with unequal variances. All tests were two-tailed, and statistical significance was set at *p* < 0.05.

## 3. Results

### 3.1. Analysis of Heptachlor Content in PSPFs

Table 1 summarizes the cultivation fields and heptachlor concentrations of PSPFs produced on different manufacturing dates (Supplementary Figure S1). The heptachlor concentrations varied depending on the production date, and samples No. 1, 3, and 4 exceeded the Japanese regulatory limit for animal feed (0.02 μg/g). In contrast, all samples (No. 7–11) derived from pumpkins cultivated in Air Water Group’s contracted fields exhibited heptachlor concentrations below the regulated threshold.

### 3.2. Nutritional Composition Analysis of PSPFs

The results of the nutritional composition analysis of PSPFs are presented in Table 2, and the fatty acid profile is shown in Table 3. PSPFs were rich in fatty acids, likely derived from pumpkin seeds, with a notably high proportion of unsaturated fatty acids.

### 3.3. Analysis of β-Carotene Content in PSPFs

Table 4 presents the manufacturing dates, cultivation fields of the raw pumpkins, and β-carotene concentrations for 11 different production lots of PSPFs (Supplementary Figure S2).

### 3.4. Feeding Trial in Cattle (Pilot Study)

**Figure 1:**
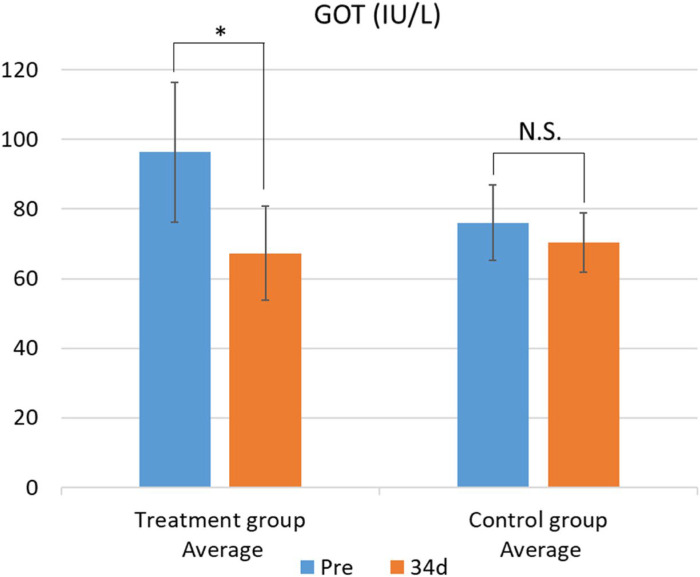
Changes in plasma GOT levels between the treatment and control groups. Data are presented as mean ± SD. Statistical significance was determined using Welch’s *t*-test; *p* < 0.05 was considered significant. Mean plasma GOT concentrations before (Pre) and after (34 d) the feeding trial in the group fed pumpkin seed pulp flakes (left) and the control group (right). * *p* < 0.05. N.S., not significant (*p* ≥ 0.05). GOT, glutamic oxaloacetic transaminase.

**Figure 2:**
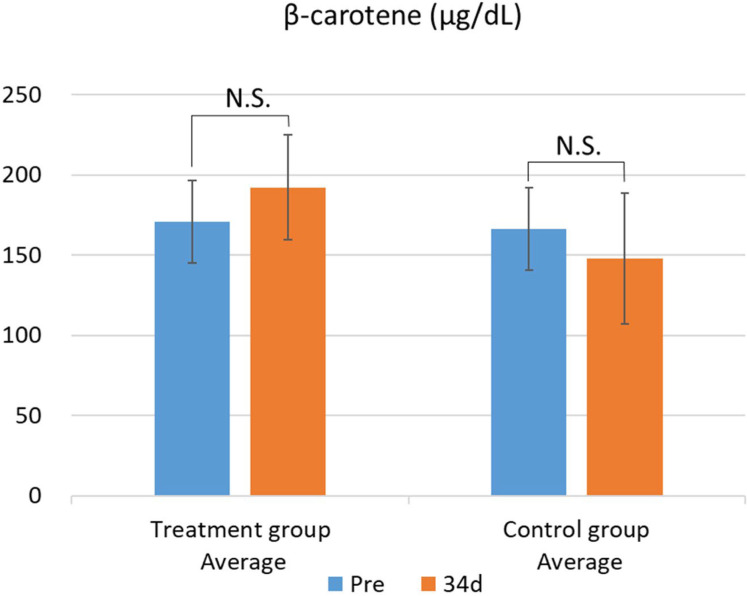
Changes in plasma β-carotene levels between the treatment and control groups. Data are presented as mean ± SD. Statistical significance was determined using Welch’s *t*-test; *p* < 0.05 was considered significant. Mean plasma β-carotene concentrations before (Pre) and after (34 d) the feeding trial in the group fed pumpkin seed pulp flakes (left) and the control group (right). N.S., not significant (*p* ≥ 0.05).

**Figure 3:**
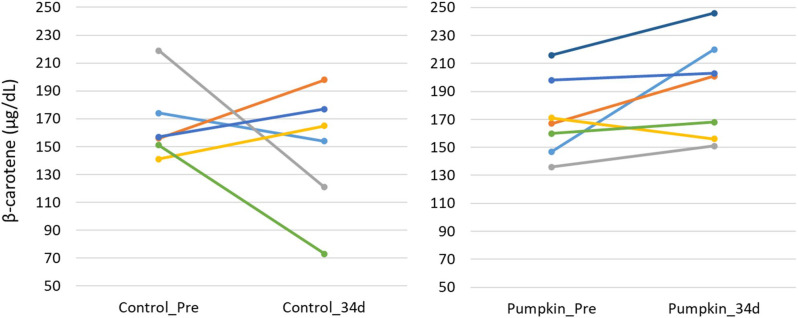
Changes in plasma β-carotene concentration. Changes in plasma β-carotene concentrations before (control_Pre) and after 34 days (control_34d) of the feeding trial in the control group (**left**), and before (pumpkin_Pre) and after 34 days (pumpkin_34d) in the group fed pumpkin seed pulp flakes (**right**).

The results of the blood biochemical analysis are shown in Table 5 and Table 6. In the test group, GOT values significantly decreased (Figure 1). This difference was confirmed by the F-test followed by Welch’s *t*-test, with a *p*-value less than 0.05. In contrast, no significant changes were observed in the other parameters. While there was no significant difference in blood β-carotene levels, the test group showed a tendency for increased blood β-carotene concentrations compared with that of the control group (Figure 2 and Figure 3).

Feedback from the farm where the trial was conducted indicated that the dried PSPFs were easy to store long-term, mix into concentrated feed, and feed to cattle. The flakes were highly palatable, and the cattle consistently consumed all of their feed.

## 4. Discussion

This study was conducted as a pilot investigation in preparation for a large-scale feeding trial, aiming to evaluate the effects of pumpkin seed pulp, which is already used as a dietary supplement, on physiological parameters in cattle. The results suggest that pumpkin seeds and fibrous pulp, which are typically discarded as wastes, can be processed into a form suitable for livestock feed to serve as a 100% natural β-carotene additive. Amid global population growth and the escalating impacts of climate change, the food supply system is facing serious challenges. In response to sustainability concerns, previous studies have explored the use of pumpkin waste as livestock feed [22]. These efforts have primarily focused on its protein and lipid content. In contrast, the present study investigates the potential of pumpkin waste as a source of β-carotene, thereby offering a novel perspective to this line of research.

Here, we processed seed and stringy pulp waste from Hokkaido-grown pumpkins by crushing and drying them into flakes. We then analyzed the samples for residual pesticide levels (heptachlor), nutritional composition, and fatty acid and β-carotene contents.

Our analysis revealed that residual heptachlor concentrations varied depending on the cultivation field, regardless of the production period. This variation is presumed to result from the presence or absence of residual heptachlor in the soil where the pumpkins were grown. Notably, all production lots derived from pumpkins cultivated in contract fields managed by the Air Water Group complied with the Japanese regulatory limit for animal feed. These findings suggest that by tracing the cultivation fields of the raw pumpkins, it is possible to control the safety of the final product with respect to residual pesticides, which may become concentrated during the drying process. To further enhance product safety, it is recommended to conduct pre-cultivation soil analyses for residual pesticides and confirm the absence of heptachlor in the designated fields.

The produced PSPFs contained an average of 7100 μg/100 g of β-carotene. However, many of the samples had been stored for over one year since production and factors, such as storage conditions in paper bags, may have contributed to a decrease in β-carotene content compared to immediately after production. At the time of manufacture, the flakes contained approximately 17,000 μg/100 g of β-carotene. These findings suggest that the β-carotene concentration in PSPFs may decrease by approximately half after one year of storage. To minimize this degradation, improvements in storage conditions, such as airtight sealing, light shielding, and control of temperature and humidity, are necessary. Although PSPFs storage conditions could be further improved, our analysis revealed high concentrations of β-carotene in PSPFs.

Our findings suggest that PSPFs have the potential to serve as a 100% natural source of β-carotene for livestock. In recent years, increasing reliance on preserved feed has raised concerns about β-carotene deficiency in livestock [13,14,15]. Such deficiencies have been associated with adverse effects, including reduced reproductive performance, impaired immune function, and diminished milk quality, all of which may significantly impact the productivity of livestock systems [5,8,11,12,37,38,39]. Some livestock producers currently use synthetic β-carotene as a feed additive to prevent β-carotene deficiency. However, processing unused parts of pumpkins into feed allows livestock to obtain sufficient β-carotene from an entirely natural, high-quality source.

Since PSPFs are made entirely from pumpkin, they are likely highly palatable to cattle, which was confirmed in the present pilot trial. In addition, our analysis revealed that PSPFs are rich in fatty acids, particularly unsaturated fatty acids, derived from crushed pumpkin seeds. The health benefits of fatty acids derived from pumpkin seeds have been reported in previous studies [40,41]. Further compositional analyses and feeding trials are needed to evaluate potential additional positive effects of fatty acids on cattle.

A pilot study was conducted using 13 cattle, with 7 assigned to the treatment group and 6 to the control group. In the treatment group, a trend toward increased plasma β-carotene concentrations was observed. Notably, freshly manufactured PSPFs were used in the feeding trial. The increase in plasma β-carotene levels in the treated cattle is presumed to result from the absorption of β-carotene contained in the flakes. These findings support the potential of PSPFs as a promising source of β-carotene for cattle.

Maintaining adequate plasma β-carotene concentrations in cattle has been reported to enhance immune function and improve reproductive performance [4,5,6,7,8,9,10,11,12]. In particular, sustaining plasma β-carotene levels above 200 μg/dL has been shown to promote postpartum recovery of reproductive function and increase conception rates [8]. In our pilot study, four out of seven cattle in the treatment group exhibited plasma β-carotene concentrations exceeding 200 μg/dL following the PSPFs feeding period. These results suggest that supplementing cattle feed with PSPFs may improve plasma β-carotene levels and contribute to enhanced reproductive performance.

The importance of β-carotene in bovine reproduction has been documented in numerous studies. Studies on Hanwoo cattle have shown that feeding fat-coated β-carotene increases blood β-carotene levels and significantly improves pregnancy rates after embryo transfer [42]. Similarly, Japanese Black cattle with high plasma β-carotene content have high progesterone secretion and superior reproductive function [43].

The improvement in reproductive function associated with β-carotene is presumed to involve the following three mechanisms:

(1) Vitamin A, which is converted from β-carotene in the body, is essential for maintaining ovarian function, follicular maturation, corpus luteum formation, and endometrial health. A deficiency in vitamin A can lead to reproductive disorders, such as delayed estrus, infertility, and miscarriage.
(2) β-carotene is a potent antioxidant that protects ovarian and uterine cells from oxidative stress. Oxidative stress can impair oocyte quality and hinder embryo implantation; therefore, its mitigation by β-carotene may contribute to improved conception rates.
(3) In cattle with higher blood β-carotene concentrations, corpus luteum development is enhanced, and the secretion of progesterone, critical for the maintenance of pregnancy, is increased and stabilized, thereby promoting embryo implantation and retention.

Additionally, the test group exhibited a significant reduction in serum GOT levels, a biomarker of hepatic injury. A similar trend has been previously reported [44], where dietary supplementation with pumpkin seed cake led to decreased GOT activity in cows. Furthermore, seed protein derived from pumpkin (*Cucurbita pepo*) has been shown to ameliorate liver dysfunction, including elevated GOT levels, induced by an unbalanced diet [45,46]. Although PSPFs developed in the present study were derived from *Cucurbita maxima*—a species distinct from those used in the aforementioned studies—the possibility of a comparable hepatoprotective effect cannot be excluded. To confirm this hypothesis, further investigations are needed, including comparative analyses of the seed protein profiles of *Cucurbita pepo* and *Cucurbita maxima* squash, as well as veterinary clinical trials in cattle.

## 5. Conclusions

This study evaluated the potential of using pumpkin seed and stringy pulp waste from Hokkaido-pumpkin processing plants as a source of β-carotene for livestock. Crushing and drying the seeds and stringy pulp together prevented the emergence of wild pumpkins and made the waste suitable for feed. Residual pesticide (heptachlor) analysis indicated variability based on pumpkin cultivation site. However, PSPFs produced from pumpkins cultivated in fields contracted by the Air Water Group met the domestic feed standards in Japan. The produced PSPFs contained high levels of β-carotene, and feeding trials in Japanese Black cows showed a tendency for increased blood β-carotene levels and significant improvement in GOT values. Importantly, no adverse effects were observed in the pilot study, indicating the safety of PSPFs as feed.

These findings suggest that PSPFs have the potential to serve as a 100% natural source of β-carotene for cattle. The addition of high-quality β-carotene to cattle feed may contribute to improvements in immune function, reproductive performance, and hepatocellular protection. Furthermore, the seeds and fibrous pulp of *Cucurbita maxima*, which serve as the raw materials for PSPFs, are generally not consumed by humans and therefore do not compete with conventional human food resources.

Amid increasing global food insecurity driven by climate change, using underutilized pumpkin components for β-carotene supplementation in cattle represents a promising strategy for both the upcycling of agricultural by-products and enhancement of livestock productivity. Further research is needed to evaluate the health effects of PSPFs-derived β-carotene in cattle and elucidate the underlying mechanisms.

We demonstrated the potential of using pumpkin seed and stringy pulp waste, typically treated as industrial waste, as livestock feed. This initiative has the potential to have a positive impact on both pumpkin farmers and livestock farmers by increasing the value of pumpkins and providing high-quality, naturally derived feed for livestock. We will continue to promote the effective use of unused resources, such as pumpkin seeds and stringy pulp waste, contributing to the development of agriculture and livestock industries in Japan and addressing environmental issues.

## Figures and Tables

**Table 1:** Heptachlor concentrations of pumpkin seed pulp flakes.

Sample No.	Manufacturing Date	Raw Material Field	Heptachlor Concentration (μg/g)
1	11 November 2023	A·B·C	0.089
2	22 November 2023	A·C·D·E	0.008
3	2 December 2023	A·D·E	0.100
4	9 January 2024	A·D·E·F	0.069
5	20 January 2024	A·D·G	0.005
6	5 February 2024	A·B	0.015
7	7 March 2024	AW *	0.006
8	16 March 2024	AW *	<0.002
9	20 March 2024	AW *	<0.002
10	1 April 2024	AW *	0.004
11	9 May 2024	AW *	0.007

* Air Water Group contracted fields.

**Table 2:** Comprehensive nutritional analysis of pumpkin seed pulp flakes.

Category	Item	Abbreviation	Unit	Result
Proximate Composition	Dry Matter	DM	%	94.5
	Crude Protein	CP	%	21.5
	Ether Extract (Crude Fat)	EE	%	19.4
	Ash	-	%	5.5
Fiber Analysis	Neutral Detergent Fiber	NDF	%	38.3
	Acid Detergent Fiber	ADF	%	29.9
	Acid Detergent Lignin	ADL	%	11.2
Energy Evaluation	Total Digestible Nutrients	TDN	%	75.3
	Net Energy for Lactation	NEL	Mcal/kg	1.86
	Net Energy for Maintenance	NEM	Mcal/kg	1.86
	Net Energy for Gain	NEG	Mcal/kg	1.25
Mineral Analysis	Calcium	Ca	%	0.06
	Phosphorus	P	%	0.65
	Magnesium	Mg	%	0.24
	Potassium	K	%	1,93

Manufacturing date: 8 May 2024.

**Table 3:** Fatty acids in pumpkin seed pulp flakes.

Fatty Acids	Concentration (g/100 g)
Total fatty acids	18.7
Saturated fatty acids	3.8
Monounsaturated fatty acids	7.3
Polyunsaturated fatty acids	7.6
Omega-3 polyunsaturated fatty acids	0.2
Omega-6 polyunsaturated fatty acids	7.4
Omega-9 polyunsaturated fatty acids	7.2
Palmitic acid (C16:0)	2.5
Stearic acid (C18:0)	1.2
Oleic acid (C18:1 n-9)	7.2
cis-Vaccenic acid (C18:1 n-7)	0.1
Linoleic acid (C18:2 n-6)	7.4
Alpha-linolenic acid (C18:3 n-3)	0.2
Arachidic acid (C20:0)	0.1

Manufacturing date: 8 May 2024.

**Table 4:** β-carotene concentrations of pumpkin seed pulp flakes.

Sample No.	Manufacturing Date	Raw Material Field	β-Carotene Concentration (μg/100 g)
1	11 November 2023	A·B·C	4800
2	22 November 2023	A·C·D·E	6900
3	2 December 2023	A·D·E	13,000
4	9 January 2024	A·D·E·F	8100
5	20 January 2024	A·D·G	7300
6	5 February 2024	A·B	6400
7	7 March 2024	AW *	3800
8	16 March 2024	AW *	6800
9	20 March 2024	AW *	6500
10	1 April 2024	AW *	5500
11	9 May 2024	AW *	9200

* Air Water Group contracted fields.

**Table 5:** The results of the blood biochemical analysis (Treatment group).

Parameter			GOT	GGT	T-CHO	TP	ALB	A/G Ratio	BUN	GLU	NEFA	Ca	IP	Mg	3-HB	VA	βC
Unit			IU/L	IU/L	mg/100 mL	g/100 mL	g/100 mL	-	mg/100 mL	mg/100 mL	mmol/L	mg/100 mL	mg/100 mL	mg/100 mL	mmol/L	IU/dL	μg/dL
Treatment group	Pre	1	70	9	158	7.3	3.4	0.9	55	8.9	5.4	2.4	466	90	147	90	147
		2	113	26	165	7.4	3.5	0.9	59	9.4	5.9	2.3	454	90	167	90	167
		3	72	14	104	8.3	3.2	0.6	51	9.0	5.5	2.1	445	83	136	83	136
		4	113	15	91	7.1	3.3	0.9	62	9.4	5.9	2.0	337	97	171	97	171
		5	109	36	146	8.3	3.2	0.6	85	9.5	5.5	1.9	288	97	198	97	198
		6	78	15	108	6.5	2.9	0.8	51	8.1	4.8	2.9	426	97	160	97	160
		7	119	22	130	8.3	3.6	0.8	73	9.3	5.6	2.3	397	100	216	100	216
		Mean ± SD	96 ± 22	20 ± 9.1	129 ± 29	7.6 ± 0.71	3.3 ± 0.23	0.79 ± 0.13	62 ± 13	9.1 ± 0.49	5.5 ± 0.37	2.3 ± 0.33	402 ± 66	93 ± 6.0	171 ± 28	93 ± 6.0	171 ± 28
	34 d	1	42	12	137	5.4	2.7	1.0	34	6.9	4.7	1.9	296	80	220	80	220
		2	70	40	195	8.1	3.7	0.9	65	10.2	5.4	2.6	393	70	201	70	201
		3	60	16	118	8.4	3.3	0.6	49	8.9	4.5	2.2	304	80	151	80	151
		4	67	17	100	6.9	3.2	0.9	60	9.1	5.6	2.2	342	90	156	90	156
		5	88	46	164	8.4	3.1	0.6	12	67	111	9.4	6.5	1.4	209	67	203
		6	65	19	133	7.1	3.2	0.8	16	62	66	9.2	6.1	2.2	417	87	168
		7	79	26	140	8.2	3.6	0.8	17	68	47	9.4	6.2	2.6	365	100	246
		Mean ± SD	67 ± 15	25 ± 13	141 ± 31	7.5 ± 1.1	3.3 ± 0.33	0.80 ± 0.15	14 ± 2.7	61 ± 7.0	62 ± 25	9.0 ± 1.0	5.6 ± 0.76	2.2 ± 0.42	332 ± 70	82 ± 11	192 ± 35

pre, Before administration; 34 d, 34 days after administration; GOT, glutamic oxaloacetic transaminase; GGT, gamma-glutamyl transferase; T-CHO, total cholesterol; TP, total protein; ALB, albumin; A/G ratio, albumin/globulin ratio; BUN, blood urea nitrogen; GLU, glucose; NEFA, non-esterified fatty acid; Ca, calcium; IP, phosphorus; Mg, magnesium; 3-HB, 3-hydroxybutyrate; VA, vitamin A; βC, β-carotene.

**Table 6:** The results of the blood biochemical analysis (Control group).

Parameter			GOT	GGT	T-CHO	TP	ALB	A/G Ratio	BUN	GLU	NEFA	Ca	IP	Mg	3-HB	VA	βC
Unit			IU/L	IU/L	mg/100 mL	g/100 mL	g/100 mL	-	mg/100 mL	mg/100 mL	mmol/L	mg/100 mL	mg/100 mL	mg/100 mL	mmol/L	IU/dL	μg/dL
Control group	Pre	1	83	6	139	7.1	3.2	0.8	51	9.6	6.9	1.9	377	100	174	100	174
		2	72	7	144	6.8	3.4	1.0	56	9.2	6.9	2.5	408	90	156	90	156
		3	79	12	151	7.1	3.6	1.0	87	9.9	6.2	2.2	437	117	219	117	219
		4	54	17	143	7.1	3.3	0.9	52	9.4	6.2	2.1	448	87	141	87	141
		5	87	7	130	6.8	3.4	1.0	142	9.4	5.9	2.2	301	97	157	97	157
		6	81	6	151	7.2	3.4	0.9	40	9.5	6.5	2.0	306	80	151	80	151
		Mean ± SD	76 ± 12	9.2 ± 4.4	143 ± 7.9	7.0 ± 0.17	3.4 ± 0.13	0.93 ± 0.08	71 ± 38	10 ± 0.24	6.4 ± 0.41	2.2 ± 0.21	380 ± 64	95 ± 13	166 ± 28	95 ± 13	166 ± 28
	34 d	1	74	10	152	7.3	3.3	0.8	69	10.2	5.6	2.1	252	70	154	70	154
		2	72	14	144	6.6	3.3	1.0	54	9.2	6.5	2.7	327	90	198	90	198
		3	70	15	144	7.1	3.6	1.1	60	10.1	5.9	2.2	282	70	121	70	121
		4	52	12	154	7.4	3.5	0.9	88	9.3	5.8	2.1	300	77	165	77	165
		5	78	16	132	6.8	3.4	1.0	93	9.5	5.9	2.2	250	93	177	93	177
		6	76	1	150	7.3	3.5	0.9	41	9.8	5.6	2.1	267	103	73	103	73
		Mean ± SD	70 ± 9.4	11 ± 5.5	146 ± 8.0	7.1 ± 0.32	3.4 ± 0.12	1.0 ± 0.10	68 ± 20	10 ± 0.42	5.9 ± 0.33	2.2 ± 0.23	280 ± 30	84 ± 14	148 ± 45	84 ± 14	148 ± 45

pre, Before administration; 34 d, 34 days after administration; GOT, glutamic oxaloacetic transaminase; GGT, gamma-glutamyl transferase; T-CHO, total cholesterol; TP, total protein; ALB, albumin; A/G ratio, albumin/globulin ratio; BUN, blood urea nitrogen; GLU, glucose; NEFA, non-esterified fatty acid; Ca, calcium; IP, phosphorus; Mg, magnesium; 3-HB, 3-hydroxybutyrate; VA, vitamin A; βC, β-carotene.

## Data Availability

The data presented in this study are available on request from the corresponding author, E.K., due to the inclusion of analysis data from external institutions.

## Supplementary Materials

The following supporting information can be downloaded at: https://www.mdpi.com/article/10.3390/metabo15080511/s1, Figure S1: Excerpt of GC/MS Chromatogram of heptachlor, Figure S2: HPLC Chromatogram of β-carotene.
