# Correction: Nizuka et al. A Pilot Study on the Use of Pumpkin Waste as Cattle Feed. *Metabolites* 2025, *15*, 511

**DOI:** 10.3390/metabo15110718

**Published:** 2025-11-03

**Authors:** Minori Nizuka, Hironobu Ishihara, Jun Nakahigashi, Daisaku Matsumoto, Eiji Kobayashi

**Affiliations:** 1 Wellness Development Center, Air Water Inc., 1-7, Tsukisamu Higashi 2-jo 16-chome, Toyohira-ku, Sapporo 062-0052, Hokkaido, Japan; 2 Air Water Logistics Co., Ltd., 1-6, Tsukisamu Higashi 2-jo 16-chome, Toyohira-ku, Sapporo 062-0052, Hokkaido, Japan; 3 Shepherd Central Livestock Clinic Co., Ltd., 2090-1 Akasegawa, Akune-shi 899-1611, Kagoshima, Japan; 4 Kobayashi Regenerative Research Institute, LLC, 1 Chayano-cho, Wakayama-shi 640-8263, Wakayama, Japan

**Errors in Tables**

In the original publication [1], the certain numbers in Tables 5 and 6 as published were incorrectly transcribed. The correct Table 5 and Table 6 appear below. The authors state that the scientific conclusions are unaffected. This correction was approved by the Academic Editor. The original publication has also been updated.

## Figures and Tables

**Table 5:** The results of the blood biochemical analysis (Treatment group).

Parameter			GOT	GGT	T-CHO	TP	ALB	A/G Ratio	BUN	GLU	NEFA	Ca	IP	Mg	3-HB	VA	βC
Unit			IU/L	IU/L	mg/100 mL	g/100 mL	g/100 mL	-	mg/100 mL	mg/100 mL	mmol/L	mg/100 mL	mg/100 mL	mg/100 mL	mmol/L	IU/dL	μg/dL
Treatment group	Pre	1	70	9	158	7.3	3.4	0.9	15	53	55	8.9	5.4	2.4	466	90	147
		2	113	26	165	7.4	3.5	0.9	14	52	59	9.4	5.9	2.3	454	90	167
		3	72	14	104	8.3	3.2	0.6	18	55	51	9.0	5.5	2.1	445	83	136
		4	113	15	91	7.1	3.3	0.9	15	61	62	9.4	5.9	2.0	337	97	171
		5	109	36	146	8.3	3.2	0.6	14	68	85	9.5	5.5	1.9	288	97	198
		6	78	15	108	6.5	2.9	0.8	14	62	51	8.1	4.8	2.9	426	97	160
		7	119	22	130	8.3	3.6	0.8	17	63	73	9.3	5.6	2.3	397	100	216
		Mean ± SD	96 ± 22	20 ± 9.1	129 ± 29	7.6 ± 0.71	3.3 ± 0.23	0.79 ± 0.13	15 ± 1.6	59 ± 5.9	62 ± 13	9.1 ± 0.49	5.5 ± 0.37	2.3 ± 0.33	402 ± 66	93 ± 6.0	171 ± 28
	34 d	1	42	12	137	5.4	2.7	1.0	9.5	47	34	6.9	4.7	1.9	296	80	220
		2	70	40	195	8.1	3.7	0.9	13	63	65	10.2	5.4	2.6	393	70	201
		3	60	16	118	8.4	3.3	0.6	15	58	49	8.9	4.5	2.2	304	80	151
		4	67	17	100	6.9	3.2	0.9	16	62	60	9.1	5.6	2.2	342	90	156
		5	88	46	164	8.4	3.1	0.6	12	67	111	9.4	6.5	1.4	209	67	203
		6	65	19	133	7.1	3.2	0.8	16	62	66	9.2	6.1	2.2	417	87	168
		7	79	26	140	8.2	3.6	0.8	17	68	47	9.4	6.2	2.6	365	100	246
		Mean ± SD	67 ± 15	25 ± 13	141 ± 31	7.5 ± 1.1	3.3 ± 0.33	0.80 ± 0.15	14 ± 2.7	61 ± 7.0	62 ± 25	9.0 ± 1.0	5.6 ± 0.76	2.2 ± 0.42	332 ± 70	82 ± 11	192 ± 35

pre, Before administration; 34 d, 34 days after administration; GOT, glutamic oxaloacetic transaminase; GGT, gamma-glutamyl transferase; T-CHO, total cholesterol; TP, total protein; ALB, albumin; A/G ratio, albumin/globulin ratio; BUN, blood urea nitrogen; GLU, glucose; NEFA, non-esterified fatty acid; Ca, calcium; IP, phosphorus; Mg, magnesium; 3-HB, 3-hydroxybutyrate; VA, vitamin A; βC, β-carotene.

**Table 6:** The results of the blood biochemical analysis (Control group).

Parameter			GOT	GGT	T-CHO	TP	ALB	A/G Ratio	BUN	GLU	NEFA	Ca	IP	Mg	3-HB	VA	βC
Unit			IU/L	IU/L	mg/100 mL	g/100 mL	g/100 mL	-	mg/100 mL	mg/100 mL	mmol/L	mg/100 mL	mg/100 mL	mg/100 mL	mmol/L	IU/dL	μg/dL
Control group	Pre	1	83	6	139	7.1	3.2	0.8	14	55	51	9.6	6.9	1.9	377	100	174
		2	72	7	144	6.8	3.4	1.0	18	61	56	9.2	6.9	2.5	408	90	156
		3	79	12	151	7.1	3.6	1.0	15	57	87	9.9	6.2	2.2	437	117	219
		4	54	17	143	7.1	3.3	0.9	15	65	52	9.4	6.2	2.1	448	87	141
		5	87	7	130	6.8	3.4	1.0	18	62	142	9.4	5.9	2.2	301	97	157
		6	81	6	151	7.2	3.4	0.9	16	59	40	9.5	6.5	2.0	306	80	151
		Mean ± SD	76 ± 12	9.2 ± 4.4	143 ± 7.9	7.0 ± 0.17	3.4 ± 0.13	0.93 ± 0.08	16 ± 1.7	60 ± 3.6	71 ± 38	9.5 ± 0.24	6.4 ± 0.41	2.2 ± 0.21	380 ± 64	95 ± 13	166 ± 28
	34 d	1	74	10	152	7.3	3.3	0.8	11	63	69	10.2	5.6	2.1	252	70	154
		2	72	14	144	6.6	3.3	1.0	13	65	54	9.2	6.5	2.7	327	90	198
		3	70	15	144	7.1	3.6	1.1	12	58	60	10.1	5.9	2.2	282	70	121
		4	52	12	154	7.4	3.5	0.9	13	68	88	9.3	5.8	2.1	300	77	165
		5	78	16	132	6.8	3.4	1.0	16	64	93	9.5	5.9	2.2	250	93	177
		6	76	1	150	7.3	3.5	0.9	16	59	41	9.8	5.6	2.1	267	103	73
		Mean ± SD	70 ± 9.4	11 ± 5.5	146 ± 8.0	7.1 ± 0.32	3.4 ± 0.12	0.95 ± 0.10	14 ± 2.7	63 ± 3.8	68 ± 20	9.7 ± 0.42	5.9 ± 0.33	2.2 ± 0.23	280 ± 30	84 ± 14	148 ± 45

pre, Before administration; 34 d, 34 days after administration; GOT, glutamic oxaloacetic transaminase; GGT, gamma-glutamyl transferase; T-CHO, total cholesterol; TP, total protein; ALB, albumin; A/G ratio, albumin/globulin ratio; BUN, blood urea nitrogen; GLU, glucose; NEFA, non-esterified fatty acid; Ca, calcium; IP, phosphorus; Mg, magnesium; 3-HB, 3-hydroxybutyrate; VA, vitamin A; βC, β-carotene.
