# Supplementary material for: A Pilot Study on the Use of Pumpkin Waste as Cattle Feed
Source: Metabolites. 2025 Jul 31;15(8):511. doi: 10.3390/metabo15080511 (PMC12388683; doi:10.3390/metabo15080511)
Supplement: Supplementary file 1 [file metabolites-15-00511-s001.zip › metabolites-3737906-supplementary.pdf]

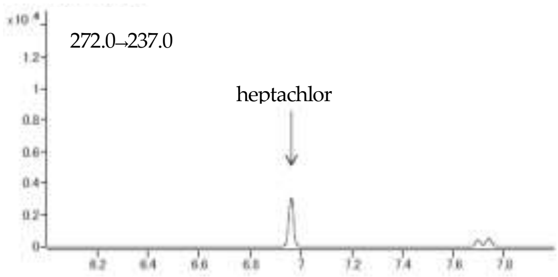

Standard Solution: heptachlor

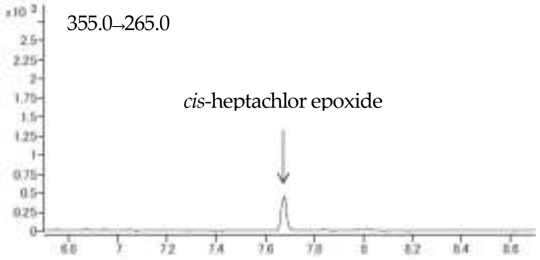

Standard Solution: *cis*-heptachlor epoxide

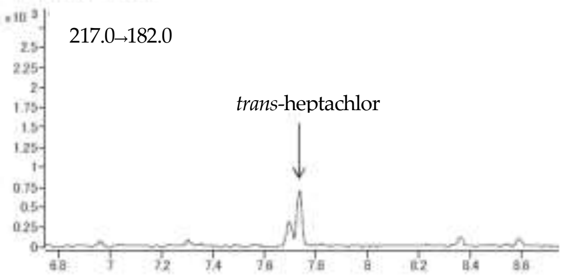

Standard Solution: *trans*-heptachlor epoxide

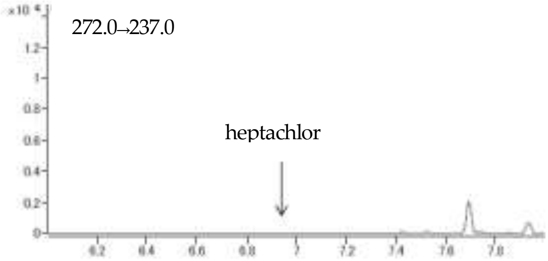

Sample Solution: Pumpkin Seed Pulp Flakes

(Manufacturing date: 2023/11/22)

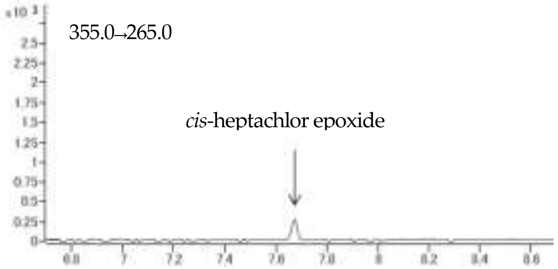

Sample Solution: Pumpkin Seed Pulp Flakes

(Manufacturing date: 2023/11/22)

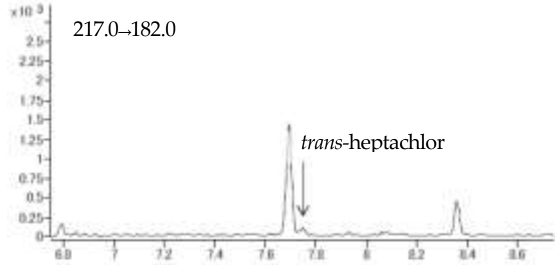

Sample Solution: Pumpkin Seed Pulp Flakes

(Manufacturing date: 2023/11/22)

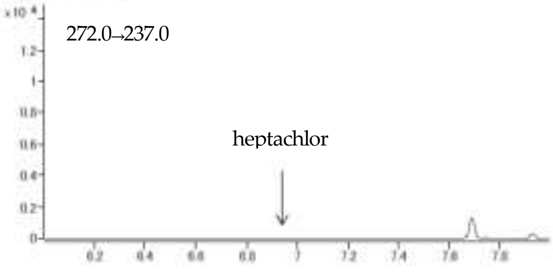

Sample Solution: Pumpkin Seed Pulp Flakes

(Manufacturing date: 2024/1/20)

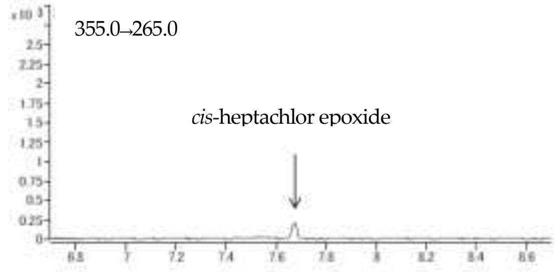

Sample Solution: Pumpkin Seed Pulp Flakes

(Manufacturing date: 2024/1/20)

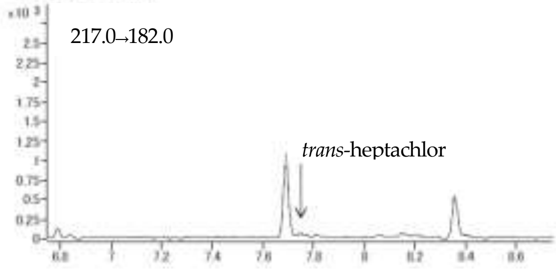

Sample Solution: Pumpkin Seed Pulp Flakes

(Manufacturing date: 2024/1/20)

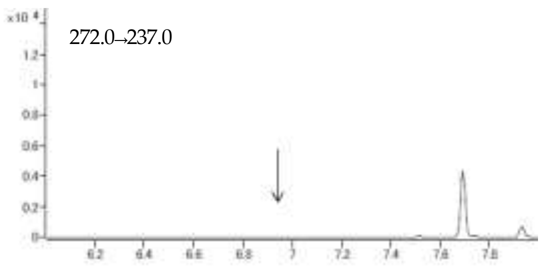

Sample Solution: Pumpkin Seed Pulp Flakes

(Manufacturing date: 2024/2/5)

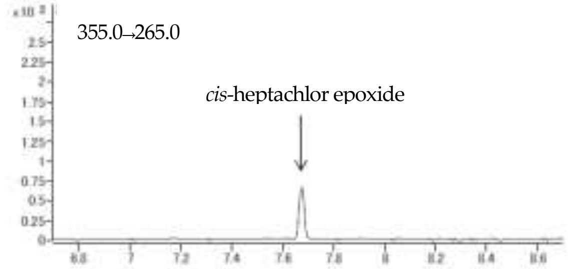

Sample Solution: Pumpkin Seed Pulp Flakes

(Manufacturing date: 2024/2/5)

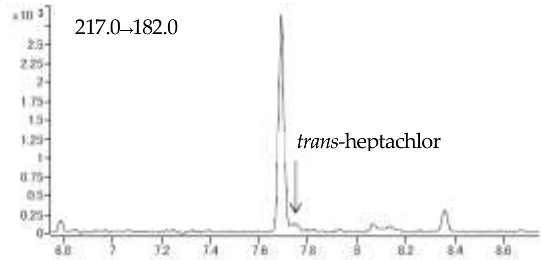

Sample Solution: Pumpkin Seed Pulp Flakes

(Manufacturing date: 2024/2/5)

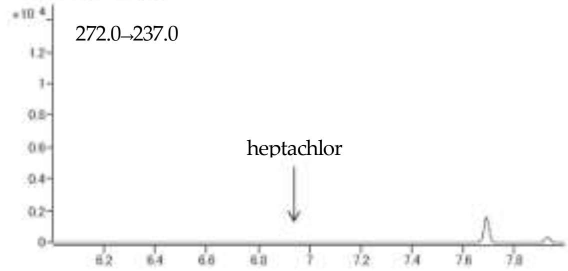

Sample Solution: Pumpkin Seed Pulp Flakes

(Manufacturing date: 2024/3/7)

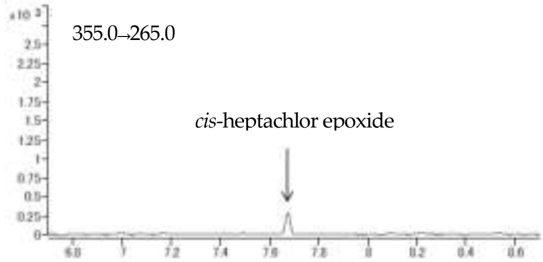

Sample Solution: Pumpkin Seed Pulp Flakes

(Manufacturing date: 2024/3/7)

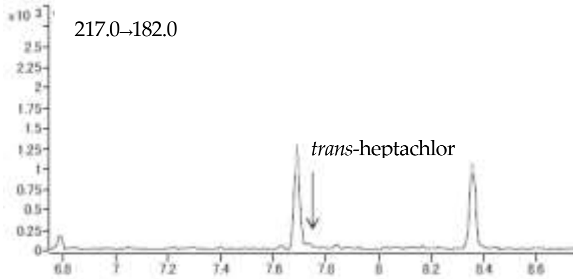

Sample Solution: Pumpkin Seed Pulp Flakes

(Manufacturing date: 2024/3/7)

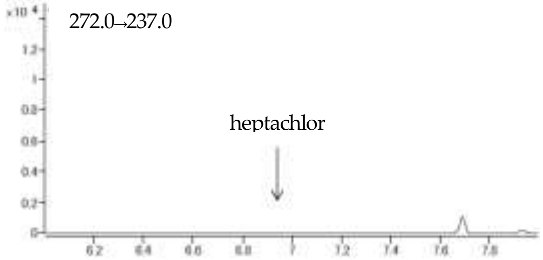

Sample Solution: Pumpkin Seed Pulp Flakes

(Manufacturing date: 2024/4/1)

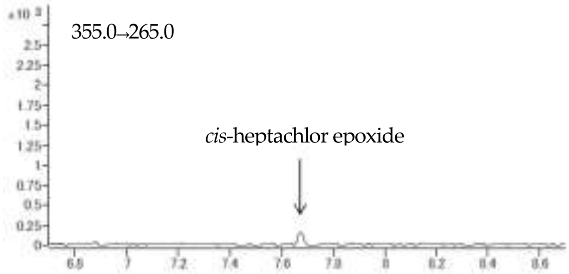

Sample Solution: Pumpkin Seed Pulp Flakes

(Manufacturing date: 2024/4/1)

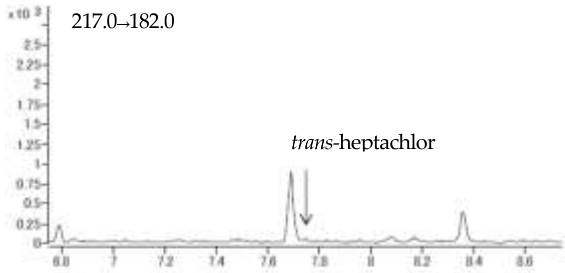

Sample Solution: Pumpkin Seed Pulp Flakes

(Manufacturing date: 2024/4/1)

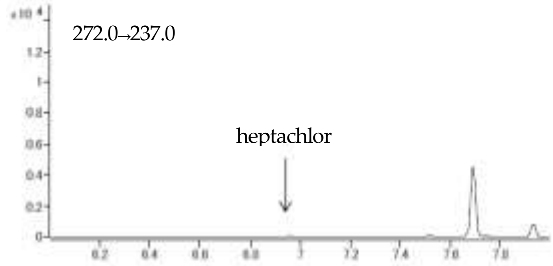

Sample Solution: Pumpkin Seed Pulp Flakes

(Manufacturing date: 2024/5/8)

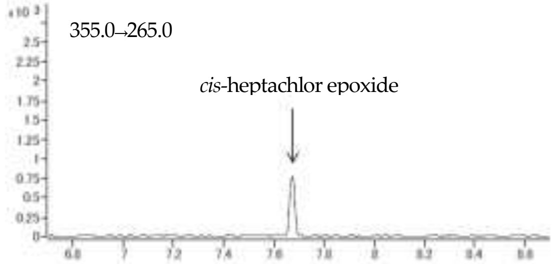

Sample Solution: Pumpkin Seed Pulp Flakes

(Manufacturing date: 2024/5/8)

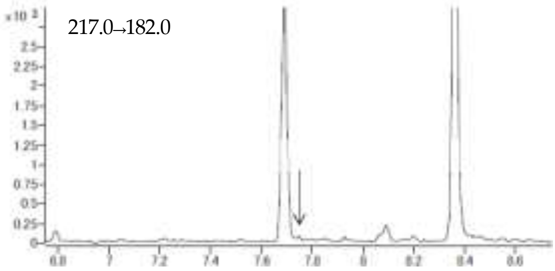

Sample Solution: Pumpkin Seed Pulp Flakes

(Manufacturing date: 2024/5/8)

Figure S1. GC/MS Chromatogram

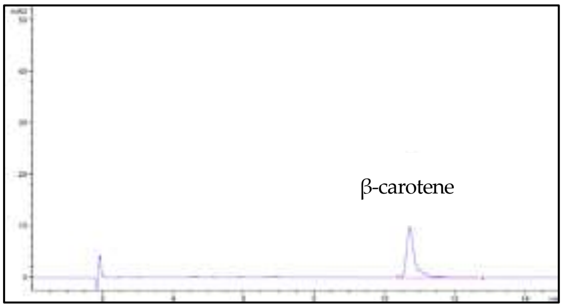

Standard Solution (β-carotene)

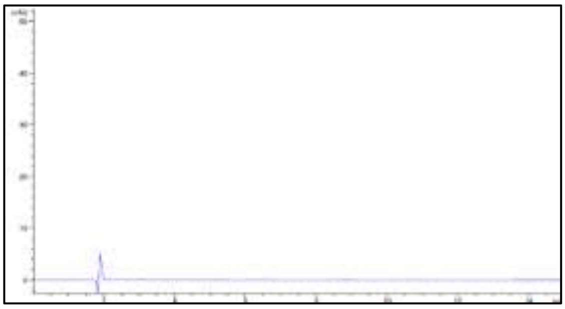

Blank Solution

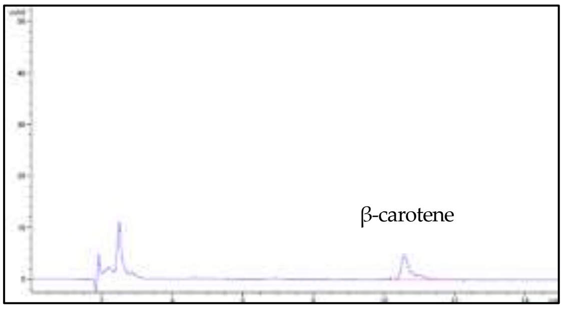

Sample Solution: Pumpkin Seed Pulp Flakes

(Manufacturing date: 2023/11/11)

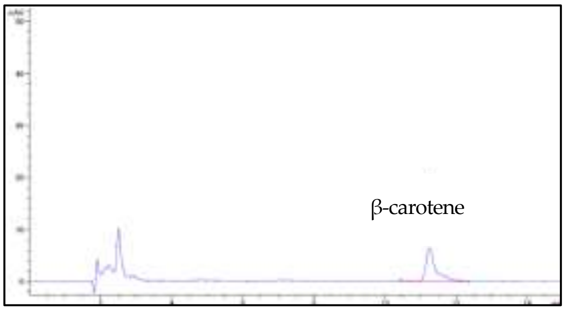

Sample Solution: Pumpkin Seed Pulp Flakes

(Manufacturing date: 2023/11/22)

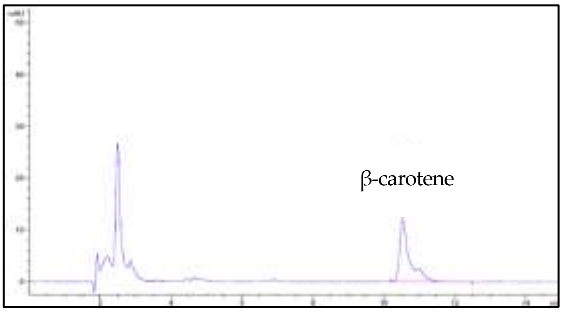

Sample Solution: Pumpkin Seed Pulp Flakes

(Manufacturing date: 2023/12/2)

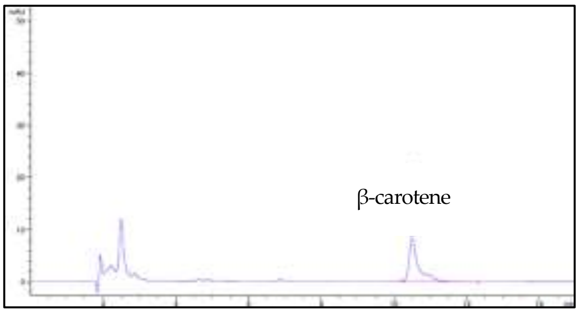

Sample Solution: Pumpkin Seed Pulp Flakes

(Manufacturing date: 2024/1/9)

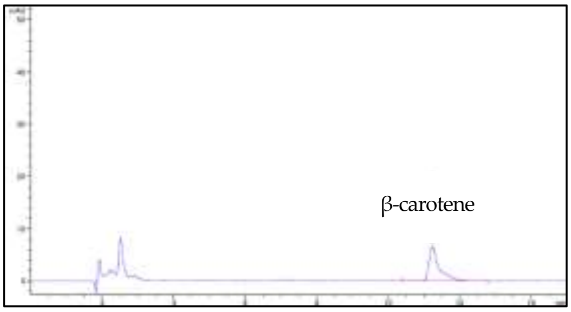

Sample Solution: Pumpkin Seed Pulp Flakes

(Manufacturing date: 2024/1/20)

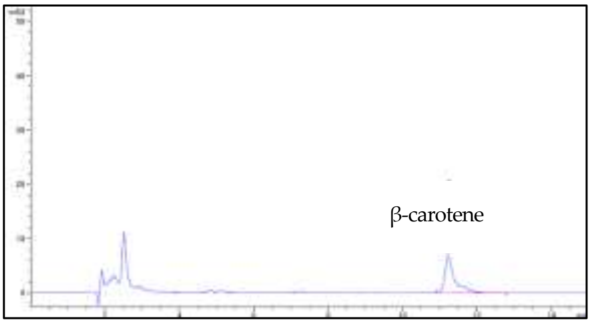

Sample Solution: Pumpkin Seed Pulp Flakes

(Manufacturing date: 2024/2/5)

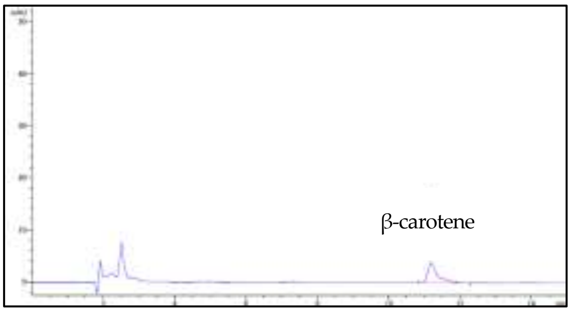

Sample Solution: Pumpkin Seed Pulp Flakes

(Manufacturing date: 2024/3/7)

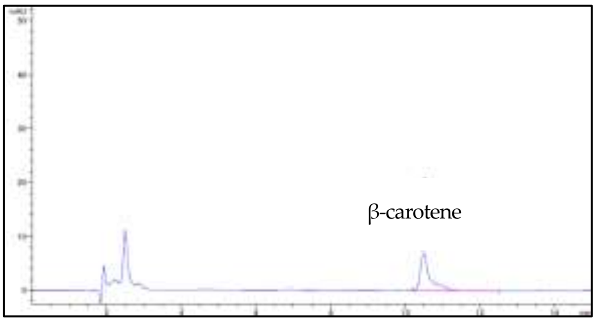

Sample Solution: Pumpkin Seed Pulp Flakes

(Manufacturing date: 2024/3/16)

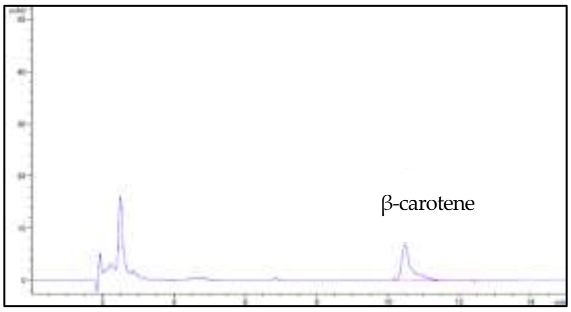

Sample Solution: Pumpkin Seed Pulp Flakes

(Manufacturing date: 2024/3/20)

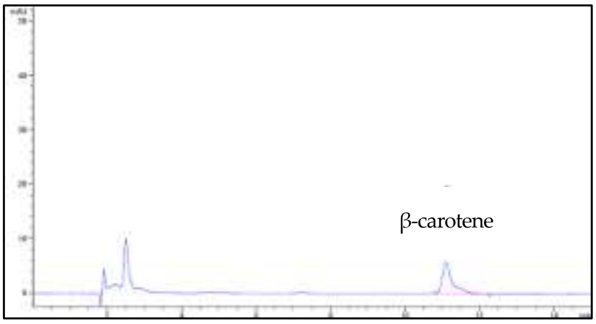

Sample Solution: Pumpkin Seed Pulp Flakes

(Manufacturing date: 2024/4/1)

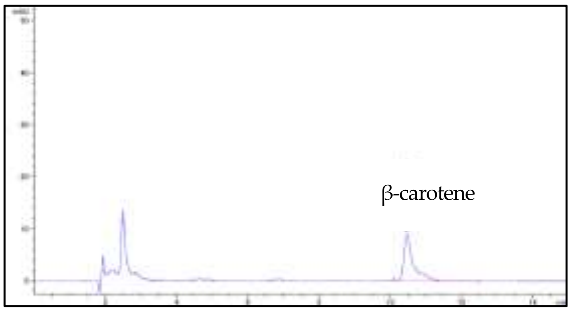

Sample Solution: Pumpkin Seed Pulp Flakes

(Manufacturing date: 2024/5/9)

Figure S2. HPLC Chromatogram
